# Effect of Meditation on Brain Activity during an Attention Task: A Comparison Study of ASL and BOLD Task fMRI

**DOI:** 10.3390/brainsci13121653

**Published:** 2023-11-29

**Authors:** Yakun Zhang, Shichun Chen, Zongpai Zhang, Wenna Duan, Li Zhao, George Weinschenk, Wen-Ming Luh, Adam K. Anderson, Weiying Dai

**Affiliations:** 1 Department of Computer Science, State University of New York at Binghamton, Binghamton, NY 13902, USAschen232@binghamton.edu (S.C.);; 2 College of Biomedical Engineering & Instrument Science, Zhejiang University, Hangzhou 310027, China; 3 National Institute on Aging, National Institutes of Health, Baltimore, MD 21225, USA; 4 Department of Psychology, Cornell University, Ithaca, NY 14853, USA; aka47@cornell.edu

**Keywords:** meditation, arterial spin labeling, functional magnetic resonance imaging, task fMRI, default mode network, dorsal attention network

## Abstract

Focused attention meditation (FAM) training has been shown to improve attention, but the neural basis of FAM on attention has not been thoroughly understood. Here, we aim to investigate the neural effect of a 2-month FAM training on novice meditators in a visual oddball task (a frequently adopted task to evaluate attention), evaluated with both ASL and BOLD fMRI. Using ASL, activation was increased in the middle cingulate (part of the salience network, SN) and temporoparietal (part of the frontoparietal network, FPN) regions; the FAM practice time was negatively associated with the longitudinal changes in activation in the medial prefrontal (part of the default mode network, DMN) and middle frontal (part of the FPN) regions. Using BOLD, the FAM practice time was positively associated with the longitudinal changes of activation in the inferior parietal (part of the dorsal attention network, DAN), dorsolateral prefrontal (part of the FPN), and precentral (part of the DAN) regions. The effect sizes for the activation changes and their association with practice time using ASL are significantly larger than those using BOLD. Our study suggests that FAM training may improve attention via modulation of the DMN, DAN, SN, and FPN, and ASL may be a sensitive tool to study the FAM effect on attention.

## 1. Introduction

Attention plays a critical role in almost every area of life, including school, work, and relationships. Attention-deficit/hyperactivity disorder (ADHD) has been linked to deficits in attention-related neural circuits [1]. Improving attention holds great promise for better daily life quality for both normal people and those coping with attention deficits. Focused attention meditation (FAM) focuses and maintains attention on the practitioner’s breathing or a small object while inhibiting distractions from internal or external resources. FAM training has been shown to correspond with improved behavioral assessments of attention, such as the attention network task (ANT) [2,3,4,5]; however, the neural basis of FAM on attention has not been thoroughly understood.

Consistent with the goal of FAM in cultivating attentional control [6], the past literature has reported activation in cortical attention networks during the meditative state [7,8,9,10]. A systematic review of electroencephalography (EEG) studies revealed that FAM was associated with alpha and theta power at the frontal electrodes compared to an eyes-closed resting state [11], indicating a state of relaxed alertness during the meditative training. The increased theta power in the frontal midline area was correlated with the time duration of meditation training and meditation deepness in experienced meditators [12,13,14]. A meta-analysis of functional magnetic resonance imaging (fMRI) studies found that FAM was associated with activation in the pre-motor cortex and right dorsolateral prefrontal cortex [15]. In contrast, FAM was associated with deactivation in the medial prefrontal cortex (mPFC), posterior cingulate cortex (PCC), and angular gyrus, which are the components of the default mode network (DMN) [15,16] and closely related to mind wandering [17].

Through repetitive activation in brain networks, FAM has been revealed to induce lasting changes (as trait characteristics) in function at the resting state and in brain structures. Meditation has been reported to induce brain morphological changes, such as increased cortical thickness [18,19,20] and more gray matter density [21], spanning several brain networks, including superior and middle frontal (frontoparietal control network, FPN), medial prefrontal cortex (default mode network, DMN), temporoparietal junction (FPN), paracentral (dorsal attention network, DAN), cingulate gyrus (salience network, SN), and superior temporal and intraparietal regions (dorsal attention network, DAN). Meditation has been associated with altered resting-state functional connectivity (rsFC) among and between DMN, DAN, FPN, and SN [16,22,23,24,25,26,27,28,29,30].

The visual and auditory oddball paradigms were frequently used in event-related potential (ERP) research to evaluate attention-related cognitive components. fMRI studies have adopted the oddball paradigms to elucidate the neural sites involved in attention, mainly in the middle frontal (dorsolateral prefrontal), medial prefrontal (deactivated), cingulate gyrus, superior temporal, paracentral, and inferior parietal regions [31,32,33,34]. Considering the overlap of brain regions involved in meditation and the oddball tasks, these tasks are well qualified to study the brain attentional changes caused by meditation. A vivid, novel oddball task was performed to evaluate the neural activation from Vipassana meditation [35]. In that study, no activation difference in attention networks was observed between experienced meditators and controls [35]. Another meditation study used the Stroop Word–Color Task, an active task to evaluate attention and impulse control, to assess the effect of meditation [36]. With a 7-day intensive Zen meditation training, novice meditators reduced activation, but more experienced meditators increased activation in several brain regions across attention networks [36]. 

Although blood oxygenation level-dependent (BOLD) fMRI has been predominantly used to study brain activation, arterial spin labeling (ASL) fMRI has been demonstrated to be capable of detecting brain activation with great sensitivity [37]. Recent technical development of dynamic ASL fMRI has been implemented with heavy background suppression, which has shown superior performance in detecting resting state networks in healthy volunteers [38] and changes in resting state networks from meditation training [28]; however, the background suppressed ASL has not been compared with BOLD for its performance in investigating the FAM effects on brain activation while performing an attentional task. The relative effect sizes will be compared. 

In this study, we aim to investigate the neural effect of a 2-month FAM training on novice meditators in a traditional visual oddball task, evaluated by both BOLD fMRI and ASL fMRI. We hypothesized that (1) FAM could change brain activation in novice meditators in the dorsolateral prefrontal, medial prefrontal, cingulate gyrus, superior temporal, paracentral, and inferior parietal regions; (2) the changes of brain activation are associated with meditation practice time; and (3) ASL fMRI is more sensitive than BOLD fMRI in the changes of activation and association with practice time.

## 2. Methods

### 2.1. Study Population

Eleven healthy participants (aged 19.09 ± 0.54 years, age range: 18 to 20 years old, 5 females, 3 left-handed) enrolled in a Binghamton University meditation course, “Meditation—Calm, Focus, and Reason”, were recruited for this study. Two of the participants had a brief meditation period, but without specific guidance for meditation in their prior yoga classes. The remaining participants had no prior experience with meditation. Therefore, all participants were novice meditators with a keen interest in practicing meditation. The study received approval from the Institutional Review Board, and all participants provided written informed consent. 

The meditation course covered various forms of meditation from different cultural backgrounds, delved into general meditation techniques, and explored the effects of meditation on physical, mental, and emotional well-being through an extensive literature review. The instructor also provided 15 min of guided meditation once or twice a week in class, exposing students to a variety of meditation styles. Participants were to choose their own object of attention during their regular meditation practice, which could include their breath, a chosen phrase, a specific point on the wall, or any other objects of their preference. During class sessions, participants were asked to sit comfortably, relax their shoulders, either close or open their eyes, concentrate on a focus point of their choice (e.g., breath), and repeatedly bring their attention back to this focus if they noticed it wandering. As part of their assignments, participants were required to engage in focused meditation for a minimum of 10 min per session, or at least five sessions per week. Additionally, they were tasked with maintaining a weekly journal where they described their experiences of meditation practice.

All eleven participants were present at the baseline MRI scans. Ten subjects attended follow-up scans after 2 months, and one female subject failed to participate and was therefore excluded from the analysis. Additionally, one male subject was excluded from task ASL analysis because no task ASL scan was performed for the subject at baseline due to the short scanning time. One female subject was excluded from task BOLD analysis because the scanned images from the subject suffered severe artifacts at the baseline. During the practice period, participants documented their practice time for each meditation session in a log file. The total practice time was calculated as the sum of all meditation practice time in minutes, including both in-class and homework practice, between the baseline and follow-up scans. 

### 2.2. Task fMRI Experimental Design

An event-related paradigm was used for a visual oddball task. A sequence of 100 visual stimuli was presented with random onsets. Visual stimuli, ‘X’ (target, 20 trails) and ‘O’ (standard, 80 trails), were presented in the center of the screen with a duration of 500 ms. The onsets of the stimuli were jittered by using a uniformly distributed inter-stimulus interval (ISI) (range: 4~5 s). Subjects were instructed to press the left button for ‘X’ and the right button for ‘O’. During the remaining time (3.5 s to 4.5 s), subjects were asked to focus on the fixation point.

### 2.3. MRI Acquisition

A GE 3T MR750 scanner (GE HealthCare, Waukesha, WI, USA) with a 32-channel receive-only phased-array head coil was used to scan the subjects, who were instructed to remain relaxed and keep awake during the scan. The anatomy of interest was defined at the beginning of scanning with a three-plane localizer. The whole brain sagittal T1-weighted magnetization prepared rapid gradient echo (MPRAGE) images were acquired in 5.5 min with the following parameters: 176 slices with the thickness = 1.0 mm and matrix size = 256 × 256, echo time (TE) = 3.42 ms, repetition time (TR) = 7 ms, inversion time (TI) = 425 ms, flip angle = 70°, field of view (FOV) = 25 cm, and receiver bandwidth (rBW) = 25 kHz. 

Resting-state dynamic pseudo-continuous arterial spin labeling (PCASL) images [38] were obtained with a 3D stack of spirals Rapid Acquisition with Refocused Echoes (RARE) readout sequence: post-labeling delay = 1.8 s, labeling duration = 2 s, FOV = 24 cm, TR = 5 s, rBW = 125 kHz, slice thickness = 4 mm, and number of slices = 40. Control and label images were obtained consecutively for each of the two spiral interleaves. Each 3D arterial spin labeling (ASL) volume required 4 TRs with a total time of 20 s. Fifty 3D ASL image volumes, along with a reference volume, were acquired in 17 min. Background suppression pulses were interleaved with the labeling pulses to suppress the magnetization of gray matter and white matter to 0.3% [38]. With background suppression, ASL signals have minimal contamination from non-neural noises, including subject motion, respiratory motion, and cardiac pulsation. The resting-state PCASL results have been reported as a separate study [28]. Lastly, subjects were scanned with a dynamic pseudo-continuous arterial spin labeling (PCASL) fMRI sequence and a BOLD fMRI sequence when performing a visual oddball task. The PCASL images during the visual oddball task were prepared with background suppression timing and acquired with the stack of spirals RARE readout sequence, in the same way as those collected during the resting state. To improve the temporal resolution, label-only single-shot (with one spiral interleave) ASL images [39] were acquired in the task ASL scan. Ninety-two label-only 3D ASL images were acquired in 8 min. BOLD fMRI images were acquired with an echo planar imaging (EPI) readout sequence: TR = 2 s, FOV = 24 cm, matrix size = 72 × 72, slice thickness = 3 mm, number of slices = 44, and interleaved slice order. Two hundred and forty BOLD fMRI images were acquired in 8 min. The order of the PCASL fMRI scan and the BOLD fMRI scan was random across these subjects.

### 2.4. Data Analyses

All PCASL images from resting-state PCASL scans and task PCASL scans were reconstructed offline using customized software implemented in MATLAB R2019a. Images were reconstructed with a standard gridding algorithm in-plane and interpolated to a matrix of 128 × 128. Mean perfusion images were reconstructed from the resting-state PCASL scans by averaging the ASL difference image between control and labeling pairs. The ASL label-only images from the task PCASL scan were reconstructed by combining the phase information from the mean perfusion images with the ASL label-only complex images. The first 3D label-only volume was discarded because of transient instabilities. Using SPM12, the 3D label-only images and reference image were realigned to correct for head motion; T1w-MPRAGE images were segmented into white matter, gray matter, and CSF images; the derived gray matter images were co-registered to the ASL reference image; and the task ASL image series were normalized to the MNI space using the transformation from the co-registered gray matter images to the MNI template. No further spatial smoothing was applied. The task BOLD fMRI images were also normalized to the MNI space using SPM12. The task BOLD images were first corrected for slice timing and head motion; the gray matter images, which were derived from the segmentation of the T1w-MPRAGE images, were co-registered to the mean motion-corrected BOLD images; and the task BOLD image series were normalized to the MNI space using the transformation from the co-registered gray matter images to the MNI template. BOLD data were smoothed using a 6 mm full-width-half-maximum (FWHM) Gaussian kernel. 

The individual activation maps were produced using the general linear model (GLM) in SPM12. The onset times of two types of stimuli, ‘X’ and ‘O’ (target and standard), and their time derivatives were modeled as regressors, convoluted with the canonical hemodynamic response function (HRF). Subsequently, the high-pass filter with a 128 s cut-off, an explicit standard mask, and no global scaling were applied. Afterwards, images of individual contrasts (‘X’, ‘O’, ‘X+O’, and ‘X−O’) were generated for each subject and each condition (baseline or follow-up).

In order to compare the changes in activation between baseline and follow-up at the group level, paired t-tests with gender and practice time as covariates were performed on individual contrast maps on a voxel-by-voxel basis. To investigate how the longitudinal changes in activation were related to meditation practice time, multiple linear regression models were performed, with the longitudinal change in activation as a dependent variable, practice time as an independent variable, and gender as a covariate. A voxel-level *p*-value of 0.005 was used to threshold the statistical maps. A cluster-level *p*-value of 0.05 was used to correct for multiple comparisons. 

Post hoc regional analyses were performed to visualize the changes in functional activation and the relationship between practice time and longitudinal activation changes. The regions of interest (ROIs) were derived as significant clusters from the voxel-based analyses. The regional activation value from the baseline/follow-up was calculated as the mean of activation over each ROI. The longitudinal activation changes in the regions of interest (ROIs) were analyzed using multiple linear regression analysis with practice time and gender as the covariates for each contrast (‘X’, ‘O’, ‘X+O’, and ‘X−O’).

To compare the sensitivity of the ASL and BOLD fMRI methods on task activation from the visual oddball tasks, we calculated the effect size for each contrast of either method. Due to the different locations activated for each contrast between these two methods, different ROIs were used to evaluate the corresponding method. The ROIs were derived from the voxel-level significant clusters for each contrast of either method. For the contrast with more than one significant cluster, the ROI was derived by combining all the significant clusters. We compared the effect sizes of ASL and BOLD on the meditation effect on functional activation using Cohen’s d, i.e., the mean of longitudinal changes in functional activation divided by their standard deviation. For the association of practice time with activation changes, the effect size was calculated as their partial Pearson correlation while controlling for the effect of gender. To compare the reliability of the effect sizes, we used the bootstrap method to randomly resample the subjects with replacement (by keeping *n* = 9) for 1000 times and calculated the average effect size for these generated samples. 

## 3. Results

### 3.1. Basic Characteristics of Subjects

Table 1 shows the basic characteristics of all the subjects, consisting of age, gender, and meditation practice time. Subjects with valid data recording of task ASL images were 19.11 ± 0.60 years old, ranging from 18 to 20, including four females. Subjects with valid data recording of task BOLD images were 19.22 ± 0.44 years old, ranging from 18 to 20, including three females.

The practice time of the ten subjects was 574.00 ± 465.55 min. No significant difference was observed between female and male participants in terms of their meditation practice times (*p* = 0.38), although two male participants practiced much longer than other participants. There is no significant difference in response time before and after meditation training (baseline: 381.24 ± 15.36 ms; follow-up: 373.23 ± 27.09 ms, *p* = 0.36). No significant difference in the percentage of correct responses was found after meditation training (baseline: 52.00% ± 19.79%; follow-up: 60.72% ± 26.28%, *p* = 0.11). 

### 3.2. Changes of Activation between Baseline and Follow-Up

**Figure 1:**
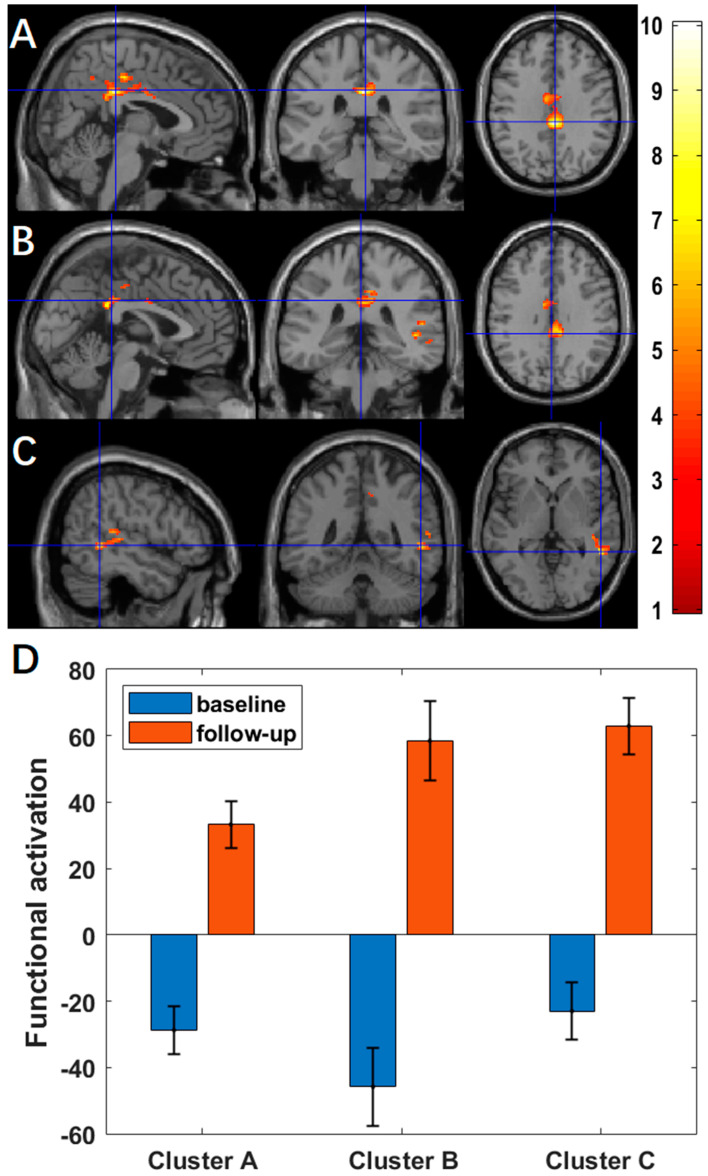
Using ASL fMRI (*n* = 9), regions overlaid on a standard brain template in which significantly increased functional activation after a 2-month meditation training was observed with a voxel-level *p*-value of 0.005 in (**A**) the middle cingulate region for the contrast of ‘X’, (**B**) the middle cingulate region, and (**C**) the superior temporal region for the contrast of ‘X+O’, and (**D**) increased functional activation for the clusters in (**A**–**C**) from the baseline to follow-up.

**Figure A1:**
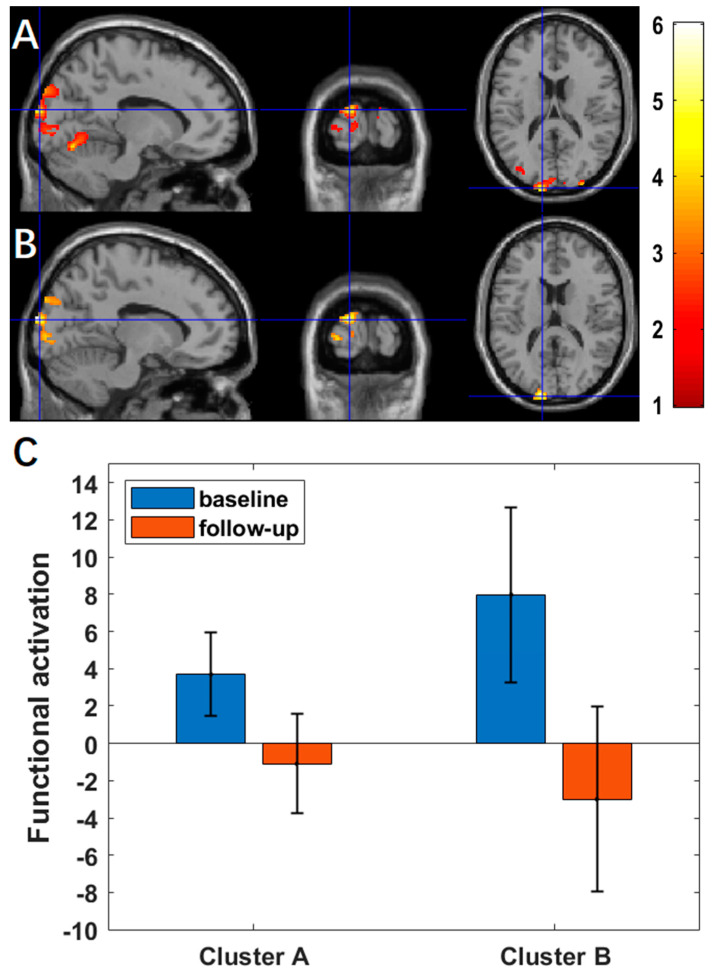
Regions overlaid on a standard brain template in which significantly decreased functional activation after a 2-month meditation training using BOLD fMRI was observed in (**A**) the occipital region for the contrast of ‘X’ (voxel-level *p* = 0.04) and (**B**) the occipital region for the contrast of ‘X+O’ (voxel-level *p* = 0.01), and (**C**) decreased functional activation for the clusters in (**A**,**B**) from the baseline to follow-up.

Using ASL fMRI with a voxel-level *p*-value of 0.005, we found significantly more activation at the two-month follow-up compared to the baseline in the middle cingulate region (cluster-level *p* < 10^−3^, Figure 1A) for the contrast of ‘X’ and in the middle cingulate region (cluster-level *p* = 0.004, Figure 1B) and superior temporal region (cluster-level *p* = 0.031, Figure 1C) for the contrast of ‘X+O’. Cluster-level statistics for the significant clusters are shown in Table 2. There were no significant changes in activation for the other contrasts. Using BOLD fMRI, no significant changes in activation were found, with a voxel-level significance threshold of 0.005. For exploratory purposes, we increased the voxel-level *p*-value by a step of 0.005 until we found a significant cluster. With a relaxed voxel-level *p*-value, we found significantly less activation at the two-month follow-up compared to the baseline in the occipital region (voxel-level *p* = 0.040, cluster-level *p* = 0.030, Figure A1A) for the contrast of ‘X’, and in the occipital region (voxel-level *p* = 0.010, cluster-level *p* = 0.048, Figure A1B) for the contrast of ‘X+O’. Cluster-level statistics for the significant clusters are shown in Table A1.

### 3.3. Relationship between Practice Time and Activation Changes

**Figure 2:**
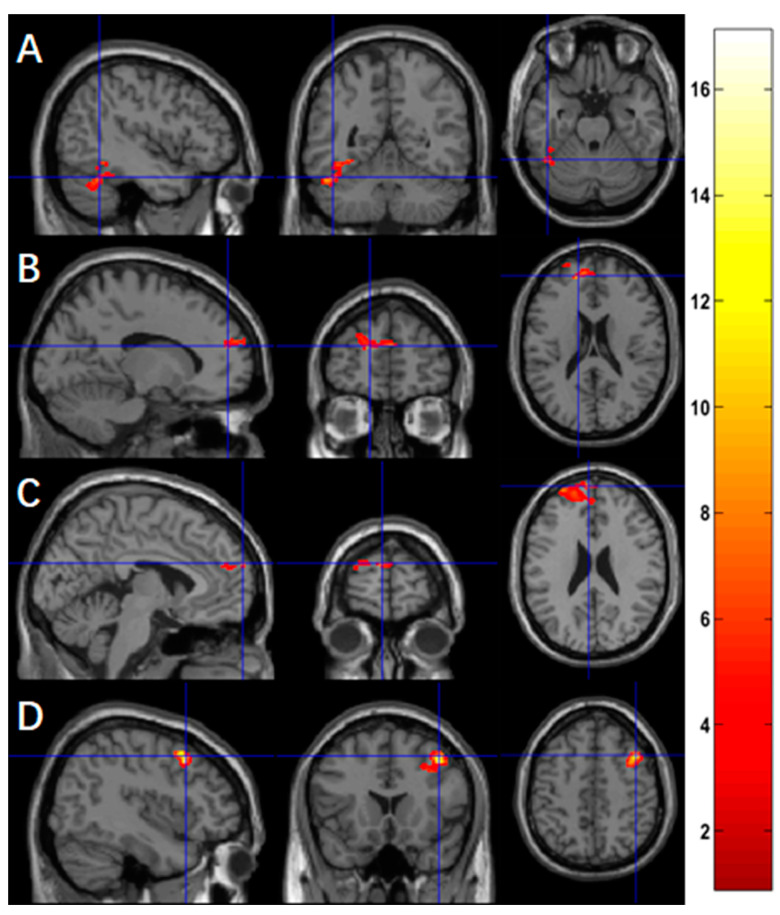
Using ASL fMRI (*n* = 9), more practice time was associated with reduced functional activation in (**A**) the left fusiform for the contrast of ‘X’, (**B**) the left superior frontal region for the contrast of ‘O’, (**C**) the superior frontal region for the contrast of ‘X+O’, and (**D**) the right middle frontal regions for the contrast of ‘X−O’.

Using ASL fMRI with a voxel-level *p*-value of 0.005, we found a significantly negative association between practice time and the changes of activation in four clusters, mainly in the left fusiform (*p* = 0.027, Figure 2A) for the contrast of ‘X’, in the left superior frontal region for the contrast of ‘O’ (*p* = 0.008, Figure 2B), in the left superior frontal region for the contrast of ‘X+O’ (*p* = 0.012, Figure 2C), and in the right middle frontal region (*p* = 0.044, Figure 2D) for the contrast ‘X−O’. Table 3 summarizes the statistics of these significant clusters.

**Figure 3:**
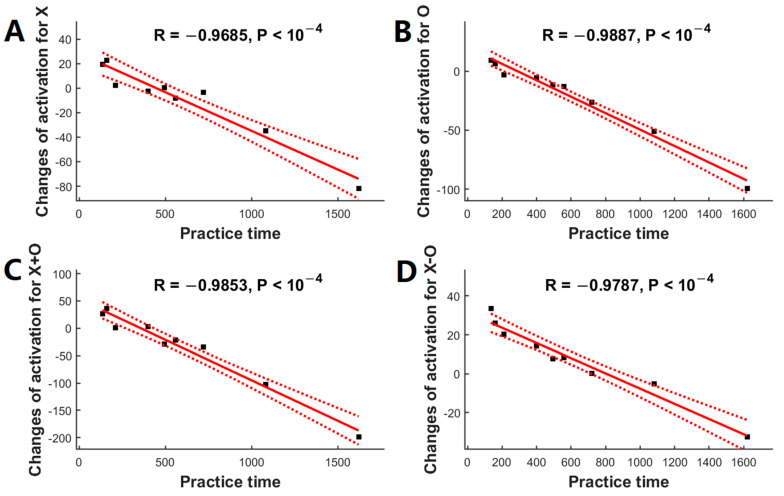
Association between the changes in functional activation (after a 2-month meditation training) and meditation practice time using ASL fMRI (*n* = 9). Meditation practice time was significantly correlated with the changes in functional activation in (**A**) the left fusiform for the contrast of ‘X’, (**B**) the left superior frontal region for the contrast of ‘O’, (**C**) the left superior frontal region for the contrast of ‘X+O’, and (**D**) the right middle frontal region for the contrast of ‘X−O’. Black squares are individual data points; solid red lines are linear regression lines; dotted red lines are 95% confidence intervals.

Post hoc analyses confirmed the significant associations between the meditation practice time and changes of regional activation after adjusting for the gender effects for the contrast of ‘X’ in the left fusiform (r = −0.9685, *p* < 10^−4^, Figure 3A), the contrast of ‘O’ in the left superior frontal region (r = −0.9887, *p* < 10^−4^, Figure 3B), the contrast of ‘X+O’ in the left superior frontal region (r = −0.9853, *p* < 10^−4^, Figure 3C), and the contrast of ‘X−O’ in the right middle frontal region (r = −0.9787, *p* < 10^−4^, Figure 3D).

**Figure 4:**
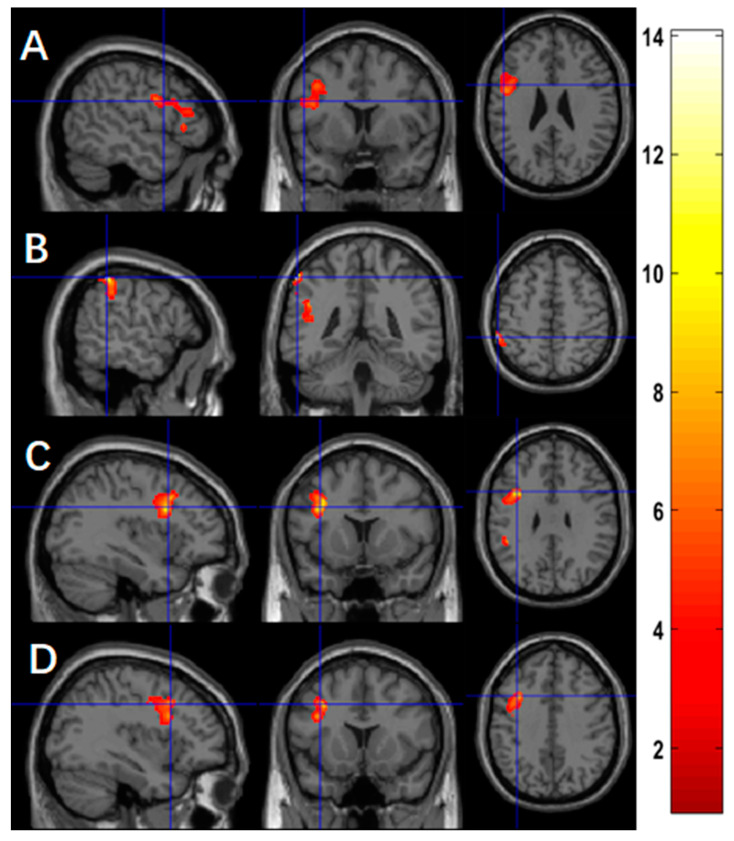
Using BOLD fMRI (*n* = 9), more practice time was associated with more increased functional activation in (**A**) the precentral region for the contrast of ‘X’, (**B**) the left inferior parietal region for the contrast of ‘O’, (**C**) the precentral region for the contrast of ‘O’, and (**D**) the precentral region for the contrast of ‘X+O’.

Using BOLD fMRI, we found a significantly positive association between practice time and the changes of activation in four clusters, mainly in the left precentral region for the contrast of ‘X’ (*p* < 10^−4^, Figure 4A), the left inferior parietal region (*p* = 0.019, Figure 4B), and the left precentral region (*p* = 0.001, Figure 4C) for the contrast of ‘O’, and the left precentral region for the contrast of ‘X+O’ (*p* < 10^−4^, Figure 4D). Table 3 summarizes the statistics of these significant clusters.

**Figure 5:**
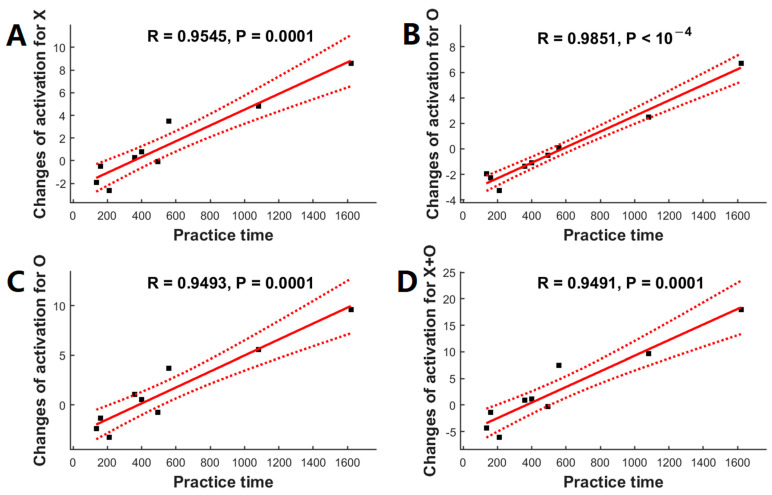
The relationship between the changes in functional activation (after a 2-month meditation training) and meditation practice time using BOLD fMRI (*n* = 9). Meditation practice time was significantly correlated with the changes in functional activation in the precentral and inferior frontal for the contrast of ‘X’ (**A**), ‘O’ (**C**), and ‘X+O’ (**D**), and left inferior parietal and superior temporal (**B**) for the contrast of ‘O’. Black squares are individual data points; solid red lines are linear regression lines; dotted red lines are 95% confidence intervals.

Post hoc analyses confirmed the significant associations between the meditation practice time and changes in regional activation for the contrast of ‘X’ in the left precentral region (r = +0.9545, *p* = 10^−4^, Figure 5A), the contrast of ‘O’ in the left inferior parietal region (r = +0.9851, *p* < 10^−4^, Figure 5B) and in the left precentral region (r = +0.9493, *p* = 10^−4^, Figure 5C), and the contrast of ‘X+O’ in the left precentral region (r = +0.9491, *p* = 10^−4^, Figure 5D). 

### 3.4. Comparison of Effect Sizes between ASL fMRI and BOLD fMRI

**Figure 6:**
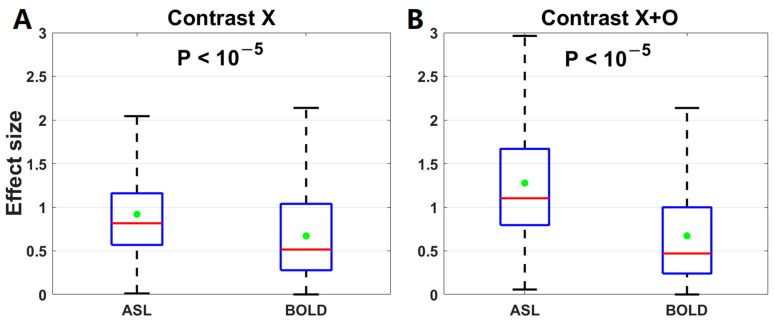
Comparison of the effect sizes of the activation changes between ASL and BOLD for (**A**) the contrast of ‘X’ and (**B**) the contrast of ‘X+O’. The horizontal red line and green dot inside each box stand for the median and mean of either fMRI method, respectively.

The effect size of the activation changes after 2-month meditation training was calculated for the contrast ‘X’ and ‘X+O’ of either method. The effect size was not compared for the other two contrasts because their activation changes were not significant using either ASL or BOLD voxel-based analyses. Using ASL fMRI, the effect size was 0.78 for the contrast of ‘X’ and 1.04 for the contrast of ‘X+O’. Using BOLD fMRI, it was 0.48 for the contrast of ‘X’ and 0.43 for the contrast of ‘X+O’. With 1000 random sampling, the effect sizes using ASL were significantly higher than those using BOLD for the contrast of ‘X’ (ASL: 0.92 ± 0.51, BOLD: 0.67 ± 0.50, *p* < 10^−5^) and the contrast of ‘X+O’ (ASL: 1.28 ± 0.66, BOLD: 0.67 ± 0.59, *p* < 10^−5^) (Figure 6).

**Figure 7:**
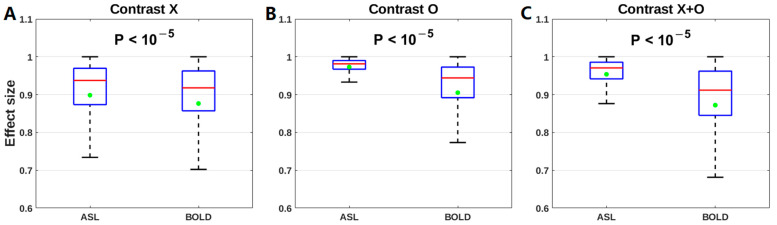
Comparison of the effect sizes of the association with practice time between ASL and BOLD for (**A**) the contrast of ‘X’, (**B**) the contrast of ‘O’, and (**C**) the contrast of ‘X+O’. The horizontal red line and green dot inside each box stand for the median and mean of either fMRI method, respectively.

The effect size of the association with practice time was calculated for each contrast ‘X’, ‘O’, and ‘X+O’ of either method. The effect size was not compared for ‘X−O’ to avoid bias because its significant correlation was found only using ASL voxel-based analysis. Using ASL fMRI, the effect size was 0.93 for the contrast of ‘X’, 0.97 for the contrast of ‘O’, and 0.97 for the contrast of ‘X+O’. Using BOLD fMRI, it was 0.90 for the contrast of ‘X’, 0.93 for the contrast of ‘O’, and 0.88 for the contrast of ‘X+O’. With 1000 random sampling, the effect sizes using ASL were significantly higher than those using BOLD for the contrast of ‘X’ (ASL: 0.90 ± 0.11, BOLD: 0.88 ± 0.15, *p* < 10^−5^), the contrast of ‘O’ (ASL: 0.97 ± 0.028, BOLD: 0.91 ± 0.13, *p* < 10^−5^), and the contrast of ‘X+O’ (ASL: 0.95 ± 0.050, BOLD: 0.87 ± 0.15, *p* < 10^−5^) (Figure 7).

## 4. Discussion

We investigated changes in brain activation while performing an attention task (the traditional oddball task) after a 2-month meditation training on novice meditators using ASL and BOLD fMRI methods. Using ASL fMRI, the meditation training increased activation in the middle cingulate (part of SN) and temporoparietal junction (part of FPN) regions; the amount of meditation practice time over a 2-month period was negatively correlated with the longitudinal changes of brain activation in the fusiform (part of visual network, VN), medial prefrontal (part of DMN), and middle frontal (part of FPN) regions. Using BOLD fMRI, no significant changes were observed after the meditation training; the amount of meditation practice time was positively correlated with the longitudinal changes of brain activation in the inferior parietal (part of DAN), dorsolateral prefrontal (part of FPN), and precentral (part of DAN) regions. For the association between meditation practice time and activation changes, the effect sizes using ASL are significantly larger than those using BOLD. Our longitudinal study extends a recent cross-sectional study that examined the attention function of experienced meditators while performing highly demanding but non-meditative tasks [35]. The experienced meditators exhibited increased activation in the DAN and deactivation in the DMN when performing a sustained attention task compared to controls, but no difference in activation when performing an oddball attention capture task. 

Insula was associated with the moment when the meditator perceived the awareness of mind wandering and pressed a button [40]. In addition to the insula, the middle cingulate was involved in modulating the frontoparietal circuit controlling hand actions [41]. While performing the oddball task, the subjects in our study were asked to press a button for the observed letter, therefore having to be aware of each moment of the task. The middle cingulate was activated more after meditation training, supporting the higher present-moment awareness of meditators [9,42]. The dorsolateral prefrontal region (DLPFC, the frontal part of FPN) was shown to be activated during sustained attention [24] and chosen as a seed for its functional connectivity during the cycle of FAM. The temporoparietal junction (the parietal part of FPN), functionally connected with DLPFC, had more activations after a 2-month meditation training in our study, explaining the increased control in goal-driven tasks after meditation training.

With more meditation practice time, participants exhibited reduced activation in the fusiform and medial prefrontal regions. The fusiform and medial frontal regions are part of VN and DMN, related to attention and self-referential processing, respectively. Less activation in the VN is consistent with reduced functional connectivity between the attention network and VN during the resting state in the same cohort [28] and during the meditative state in more experienced meditators [24]. Our results suggest that meditators may devote less attentional resources to the visual processing domain and therefore allow more efficient attention allocation during a task. Mind wandering, intended for inhibition during FAM, has been associated with activity in the DMN [17]. Reduced activity in the DMN has been reported during meditation practice in experienced meditators relative to nonmeditators [16,43]. Our findings demonstrated that reduced DMN activity during meditation practice can be extended to brain activity while performing the attention task, suggesting the role of meditation in suppressing the DMN activity and mind wandering in daily life activities. By contrast, we did not observe significant behavioral (response time and correct responses) differences on the attention task after meditation training, although the magnitudes of response time and correct responses are slightly better. This indicates that behavioral measures are less sensitive, at least compared to brain activation measures, requiring a larger sample size to see the effect.

More meditation practice time was associated with increased activation in the inferior parietal, dorsolateral prefrontal, and precentral (premotor) regions, belonging to the posterior part of DAN, frontal part of FPN, and frontal eye part of DAN, respectively. These regions were consistent with the activated sites from a meta-analysis of functional imaging during FAM meditation [15]. These activated sites are frequently activated in task-relevant studies requiring voluntary regulation of attention and monitoring of performance [44,45,46]. The involvement of those attention and executive areas is in line with the goal of meditation in sustaining attention with a range of regulatory demands.

After a 2-month meditation training, brain activation while performing an attention task changes in the SN, FPN, DAN, and DMN, consistent with brain networks involved in long-term modulation of meditation [47]. These brain networks shaped via meditation also support recent theoretical models of meditation [48,49]. As suggested by these models, as a situation demands, the FPN is rapidly and flexibly coupled with the DMN and other attention networks for contextually appropriate engagement and disengagement; therefore, meditation may shape the practitioner’s mind to a tranquil or still status to skillfully gain volitional control, flexibility, and meta-awareness over mind wandering and associated DMN activities. 

Across all contrasts, we found a significantly higher effect size for the association between changes in task activation and meditation practice time using ASL compared to BOLD, although they activated different regions. The higher effect size of ASL vs. BOLD is consistent with the higher sensitivity of ASL in characterizing the resting state functional connectivity using the same subjects [27,28]. The improved effect size of ASL may benefit from its minimal susceptibility to motion and physiological noises because of the adoption of heavy background suppression in its pulse sequence [38,39]. 

Our study has limitations. We had a small sample size and different variations of focused attention meditation. However, using ASL fMRI, we observed a trend that FAM can increase brain activity in the SN and FPN when performing an attention task. Our preliminary study suggested that ASL may be a sensitive tool to study the meditation effect on a cognitive task. Another limitation of the study is that we did not include a control group. However, the longitudinal changes of brain activation in the DMN, DAN, and FPN were correlated with meditation practice time, which is consistent with the results from a meta-analysis during meditation. The association of longitudinal changes in brain activation with meditation practice time suggests that the activation changes are caused by meditation practice itself rather than expectancy bias. A large sample size with a control group is warranted to confirm the power of ASL and the practice time correlation with task activation.

## 5. Conclusions

Our study suggests further investigation into the power of the dynamic ASL technique in investigating the effect of FAM on attention. Two-month FAM training modulates the functional activity in DMN, DAN, and FPN when subjects perform an attentional task. If confirmed in large studies, our study reveals the neural basis of FAM on improving attention and indicates potential clinical benefits of FAM to ADHD populations.

## Figures and Tables

**Table 1:** Basic characteristics and meditation practice time of subjects.

Subject ID	Age (Years)	Gender	Practice Time (Minutes)
1	19	Female	135
2	20	Male	1080
3	19	Male	1620
4	19	Male	400
5 ^a^	19	Male	360
6 ^b^	19	Female	720
7	18	Male	160
8	20	Male	495
9 ^ab^	19	Female	N/A
10	19	Female	210
11	19	Female	560

^a^ stands for the subjects who were excluded from task ASL analysis because no task ASL was scanned for subject 5 at the baseline, and subject 9 withdrew from the follow-up scan and so the practice time is shown as “N/A”. ^b^ stands for the subjects who were excluded from task BOLD analysis because task BOLD suffered severe artifacts for subject 6 at the baseline, and subject 9 withdrew from the follow-up scan.

**Table 2:** Clusters showing significant increased functional activation after a 2-month meditation training.

	Cluster-Level *p*-Value	N Voxels	Peak-t	Peak-t MNI Coordinate	Anatomical Locations		%Clusters	%Region
ASL: Increased activation for ‘X’	<10^−3^	760	9.54	4, −32, 34	Limbic System	Cingulum_Mid_R	61.18	21.11
						Cingulum_Mid_L	22.24	8.71
						Cingulum_Post_L	4.08	6.70
						Cingulum_Post_R	3.68	8.36
						Cingulum_Ant_L	0.79	0.43
						Cingulum_Ant_R	0.13	0.08
					Parietal Lobe	Precuneus_R	7.11	1.65
						Paracentral_Lobule_L	0.26	0.15
					Frontal Lobe	Supp_Motor_Area_R	0.39	0.13
						Supp_Motor_Area_L	0.13	0.05
ASL: Increased activation for ‘X+O’	0.004	444	7.07	0, −38, 28	Limbic System	Cingulum_Mid_R	67.12	13.53
						Cingulum_Mid_L	12.84	2.94
						Cingulum_Post_L	7.88	7.56
						Cingulum_Post_R	4.28	5.67
						Cingulum_Ant_L	1.35	0.43
					Parietal Lobe	Precuneus_R	6.53	0.89
	0.031	304	8.06	52, −46, 0	Temporal Lobe	Temporal_Sup_R	54.28	5.25
						Temporal_Mid_R	45.39	3.13
					Parietal Lobe	SupraMarginal_R	0.33	0.05

**Table 3:** Clusters showing significant association between meditation practice time and longitudinal changes in functional activation.

	Cluster-Level *p*-Value	N Voxels	Peak-t	Peak-t MNI Coordinate	Anatomical Locations		%Clusters	%Region
ASL: Association with practice time for ‘X’	0.027	292	9.74	−44, −52, −24	Occipital Lobe	Fusiform_L	50.68	6.41
					Temporal Lobe	Temporal_Inf_L	23.63	2.16
					Cerebellum	Cerebelum_Crus1_L	22.60	2.54
						Cerebelum_6_L	3.08	0.53
ASL: Association with practice time for ‘O’	0.008	332	10.02	−14, 52, 20	Frontal Lobe	Frontal_Sup_L	55.42	5.11
						Frontal_Sup_Medial_L	31.33	3.48
						Frontal_Mid_L	6.02	0.41
						Frontal_Sup_Medial_R	5.12	0.80
					Limbic System	Cingulum_Ant_R	2.11	0.53
ASL: Association with practice time for ‘X+O’	0.012	321	8.62	−4, 64, 24	Frontal Lobe	Frontal_Sup_L	54.52	4.86
						Frontal_Sup_Medial_L	31.46	3.38
						Frontal_Sup_Medial_R	5.61	0.84
						Frontal_Mid_L	2.80	0.19
					Limbic System	Cingulum_Ant_R	5.61	1.37
ASL: Association with practice time for ‘X−O’	0.044	250	16.43	42, 18, 48	Frontal Lobe	Frontal_Mid_R	100.00	4.90
BOLD: Association with practice time for ‘X’	<10^−4^	912	8.93	−44, 32, 12	Frontal Lobe	Precentral_L	42.21	10.92
						Frontal_Inf_Tri_L	29.71	10.72
						Frontal_Inf_Oper_L	14.91	13.10
						Frontal_Mid_L	12.72	2.39
						Rolandic_Oper_L	0.44	0.40
BOLD: Association with practice time for ‘O’	0.019	452	13.31	−56, −42, 52	Parietal Lobe	Parietal_Inf_L	39.82	7.36
						SupraMaginal_L	25.44	9.16
					Temporal Lobe	Temporal_Sup_L	24.34	4.79
					Frontal Lobe	Rolandic_Oper_L	10.40	4.75
	0.001	711	10.43	−36, 12, 30	Frontal Lobe	Precentral_L	48.10	9.70
						Frontal_Inf_Oper_L	24.19	16.57
						Frontal_Mid_L	22.78	3.33
						Frontal_Inf_Tri_L	4.36	1.23
						Rolandic_Oper_L	0.56	0.40
BOLD: Association with practice time for ‘X+O’	<10^−4^	783	8.97	−32, 14, 38	Frontal Lobe	Precentral_L	53.77	11.94
						Frontal_Inf_Oper_L	21.71	16.38
						Frontal_Mid_L	18.39	2.96
						Frontal_Inf_Tri_L	5.49	1.70
						Rolandic_Oper_L	0.64	0.51

## Data Availability

Raw data were acquired with the GE 3T MR750 scanner. MRI reconstruction software belongs to the vendor’s proprietary product. Derived data will be provided upon direct request. The data are not publicly available due to human subjects’ privacy concerns. All the shared data are free of identifiers that could link information to an individual, compliant with institutional and IRB policies.

## Abbreviations

ADHD	Attention-deficit/hyperactivity disorder
ANT	Attention network task
ASL	Arterial spin labeling
BOLD	blood oxygenation level-dependent
CSF	Cerebrospinal fluid
DAN	Dorsal attention network
DLPFC	Dorsolateral prefrontal cortex
DMN	Default mode network
EEG	Electroencephalography
EPI	Echo planar imaging
ERP	Event-related potential
FAM	Focused attention meditation
fMRI	Functional magnetic resonance imaging
FOV	Field of view
FPN	Frontoparietal control network
FWHM	Full width at half maximum
GLM	General linear model
HRF	Hemodynamic response function
ISI	Inter-stimulus interval
MNI	Montreal Neurologic Institute
mPFC	Medial prefrontal cortex
MPRAGE	Magnetization prepared rapid gradient echo
PCASL	Pseudo-continuous arterial spin labeling
PCC	Posterior cingulate cortex
RARE	Rapid Acquisition with Refocused Echoes
rBW	Receiver bandwidth
ROI	Region of interest
rsFC	Resting-state functional connectivity
SN	Salience network
SPM12	Statistical parametric mapping
TE	Echo time
TI	Inversion time
TR	Repetition time
VN	Visual network

## Appendix A

**Table A1:** Clusters showing significant changes in functional activation after 2-month meditation practice using BOLD fMRI.

	Cluster-Level *p*-Value	N Voxels	Peak-t	Peak-t MNI Coordinate	Anatomical Locations		%Clusters	%Region
BOLD: Decreased activation for ‘X’ (voxel-level *p* = 0.04)	0.030	2973	5.15	−12, −100, 18	Occipital Lobe	Occipital_Sup_L	16.01	34.85
						Lingual_L	14.70	20.86
						Occipital_Mid_L	12.18	11.07
						Cuneus_L	11.40	22.21
						Fusiform_L	7.13	9.18
						Occipital_Sup_R	5.58	11.75
						Lingual_R	5.58	7.22
						Cuneus_R	5.21	10.88
						Occipital_Inf_L	1.31	4.14
						Occipital_Mid_R	0.61	0.86
					Basal Ganglia	Calcarine_L	6.59	8.68
						Calcarine_R	6.36	10.16
					Temporal Lobe	Temporal_Inf_L	3.26	3.03
						Temporal_Mid_L	0.67	0.40
					Cerebellum	Cerebelum_6_L	2.72	4.78
						Cerebelum_4_5_L	0.54	1.42
						Vermis_6	0.07	0.54
					Parietal Lobe	Angular_L	0.07	0.17
BOLD: Decreased activation for ‘X+O’ (voxel-level *p* = 0.01)	0.048	692	6.04	−12, −100, 16	Occipital Lobe	Occipital_Sup_L	48.12	24.38
						Cuneus_L	24.13	10.94
						Occipital_Mid_L	18.35	3.88
						Lingual_L	1.30	0.43
					Basal Ganglia	Calcarine_L	8.09	2.48
